# Prevalence of short stature, underweight, overweight, and obesity among school children in Jordan

**DOI:** 10.1186/s12889-016-3687-4

**Published:** 2016-10-03

**Authors:** Ayman A. Zayed, Abdallah M. Beano, Faris I. Haddadin, Sohab S. Radwan, Suhaib A. Allauzy, Motasem M. Alkhayyat, Zaid A. Al-Dahabrah, Yanal G. Al-Hasan, Al-Motassem F. Yousef

**Affiliations:** 1 School of Medicine, University of Jordan, PO Box 13046, Amman, 11942 Jordan; 2 Faculty of Pharmacy, University of Jordan, PO Box 13046, Amman, 11942 Jordan; 3 Division of Endocrinology and Metabolism, Department of Internal Medicine, School of Medicine, The University of Jordan/Jordan University Hospital, PO Box 13046, Amman, 11942 Jordan

**Keywords:** Jordan, Short stature, Underweight, Overweight, Obesity, School children

## Abstract

**Background:**

The prevalence of short stature (SS) and underweight in Jordan on a national level is unknown. This study aimed to investigate, on a national level, the prevalence of short stature (SS), underweight, overweight, and obesity among school aged children in Jordan.

**Methods:**

This cross-sectional study was conducted from May 2015 to January 2016 and included 2702 subjects aged 6–17 years. Jordan was classified into 3 regions; North, Center (urban), and South (rural). Public and private schools were randomly selected from a random sample of cities from each region. The socioeconomic status of the sampling locations was assessed using several indicators including education, income, healthcare and housing conditions. For each participating subject, anthropometrics were obtained. SS, underweight, overweight and obesity were defined using Center of Disease Control’s (CDC) growth charts. Median Z-scores for each region, age and gender were calculated.

**Results:**

The Central and Northern regions enjoyed higher socioeconomic status compared to rural Southern regions. The overall prevalence of SS, underweight, overweight, and obesity were 4.9 %, 5.7 %, 17.3 %, and 15.7 %, respectively. SS and underweight were most prevalent in the rural South, while obesity was highest in the Central region. Females were more likely to be overweight, while males were more likely to be obese. Private schools had higher prevalence of obesity and overweight than public ones.

**Conclusions:**

Variations in height and weight among Jordanian school children might be affected by socioeconomic status.

## Background

It is becoming increasingly apparent that different forms of childhood malnutrition, from short stature (SS) and underweight to overweight, are growing global health concerns affecting developed and developing countries alike [1, 2]. The rapidly growing prevalence rates of these conditions have prompted escalated concerns into finding effective solutions. Their danger is further accentuated by the myriad of detrimental effects they have on the individual’s well being on the short and long run. Short stature for example, has been shown to be associated with disorders of the cardiovascular system [3]. In addition, SS in females was shown to adversely affect the health and survival of her offspring [4]. It is also more prevalent in regions with high infectious disease rates [5], and is linked to impaired host immunity [6]. Children with SS usually enroll later in school compared to their healthy peers, and achieve lower overall years of education [7]. Overall, SS is still considered to be directly the result of chronic malnutrition [8], and it may, therefore, be considered an accurate indicator of childhood health and may reflect a child’s nutritional and environmental background adequately. Hence, knowledge of the prevalence of SS can be considered a first step in addressing childhood health concerns. The prevalence of SS varies significantly worldwide. To our knowledge, the highest prevalence of SS was reported in Bangladesh, being 73.6 % in 1991 [9]. On the other hand, the lowest prevalence of SS in Australia was 0 % in 1995 [9].

Similarly to SS, underweight is also considered a worldwide disease burden [10] and is mostly correlated to under nutrition and social deprivation [11]. It has been shown to be associated with long term adverse effects on school achievement, cognitive development and general health [11]. The highest prevalence of underweight children recorded worldwide was found to be in Bangladesh, being 66.8 % in 1985 [9].

Obesity, on the other end of the spectrum, is also associated with short and long term physical and psychosocial complications, ranging from increased risk for cardio-metabolic diseases to lower self esteem and educational attainment, as well as higher unemployment rates [12, 13]. Childhood obesity is also a strong predictor of adult obesity [12, 13], and is currently, alongside childhood overweight, more prevalent in developed countries [14]. However, this might change in the future, since developing countries have had a greater relative increase in the prevalence of childhood obesity and overweight from 1990 to 2010 compared to developed countries [14].

Several studies were conducted in Jordan that started the process of establishing the prevalence of obesity and overweight. However, they were not national; their targets were one or two governorates of the country [15]. Additionally, there are no available data regarding the prevalence of SS and underweight children in Jordan. Thus, the aim of this study was to investigate, on a national level, the prevalence of SS, underweight and obesity among school children and adolescents in Jordan, and assess their association with gender, region (Northern, Central, and Southern regions), and school strata (private and public schools).

## Methods

### Subjects

After obtaining ethical approval from the scientific committee of the University of Jordan’s medical school, this nation-wide, cross-sectional study was conducted from May 2015 to January 2016 by the Endocrinology Division of the Jordan University Hospital, a tertiary medical center in Amman, Jordan. In total, 2702 children aged 6–17 years were enrolled in this study.

**Fig. 1:**
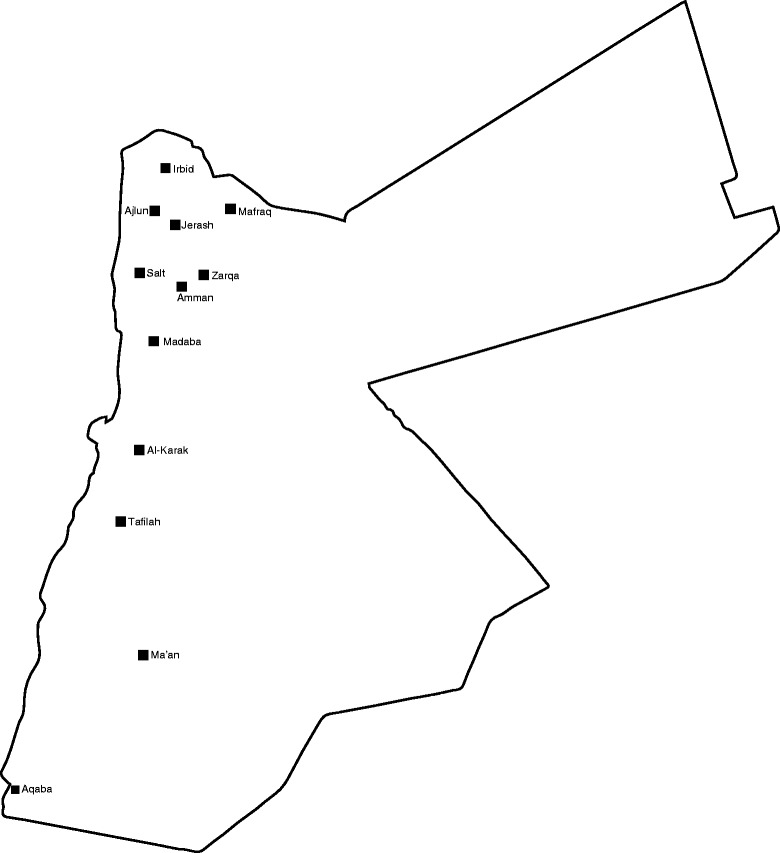
A map of Jordan outlining the cities included in the study

**Fig. 2:**
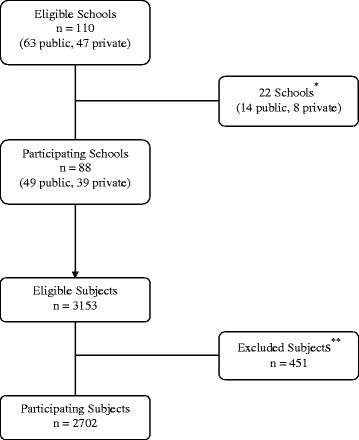
A flowchart demonstrating how schools and their students were recruited. ^*^Schools that could not participate due to logistic and administrative reasons. ^**^Subjects who were excluded because of lack of written parental consent

A random selection methodology was adopted. Jordan was classified into 3 regions; north, center, and south. All the major cities –i.e. those having a population of 100,000 or more– in each region were sorted alphabetically and given a numeric designation accordingly. Cities with odd numeric designations were selected (Fig. 1). Two school strata for each selected city were devised; public and private. Schools in either of these strata were again sorted alphabetically, and every 44th school was subsequently chosen from each stratum. All grades from the selected schools were included, and had there been more than one class division per grade, only the first class per grade –i.e. ‘Class A’– was included in the sample. The school students in their grades were sorted alphabetically and every 14th student was subsequently chosen. Due to logistic and administrative reasons among some schools, only 88 schools (49 public, 39 private) participated in the study out of 110 eligible schools (63 public, 47 private) (Table 1). The total number of participants was 3153; of those 451 could not participate because their parents did not provide written parental consent for them. Accordingly, 2702 subjects were enrolled in the study (Fig. 2).

**Table 1:** Distribution of Eligible and Participating Schools by City

Region	City/Town	Number of Schools		Number of Eligible Schools^a^		Participating Schools	
		Public	Private	Public	Private	Public	Private
*North*	Irbid^b^	509	429	11	10	9	8
	Mafraq^b^	481	43	10	1	8	1
	Jarash^b^	154	42	4	1	3	1
	Ajlun^b^	98	41	2	1	1	1
*Center*	Amman^b^	605	953	14	21	10	17
	Salt^b^	181	135	4	3	3	2
	Zarqa^b^	279	243	6	5	5	4
	Madaba^b^	99	41	2	1	2	1
*South*	Karak^c^	197	62	4	1	3	1
	Tafilah^c^	89	16	2	1	2	1
	Ma’an^c^	141	22	3	1	2	1
	Aqaba^c^	70	39	1	1	1	1

^a^Eligible; public and private, schools were selected by sorting each stratum alphabetically and every 44th school was subsequently chosen from each stratum

^b^The surrounding areas of these cities/towns were not included

^c^The surrounding areas of these cities/towns were included

### Assessments

After acquiring informed, written parental consent for all participating subjects, anthropometrics were obtained for all those enrolled by trained, senior medical students from the University of Jordan, and the chronological age was calculated by using the date of birth. Each subject’s height was measured with his or her head in a Frankfurt Plane and the occiput, shoulder, buttocks, and heels contacting a vertical board. Each subject was drawn up to full height by placing upward pressure on the mandible. Body Mass Index (BMI) was calculated as the ratio of weight to the square of height (kg/m^2^). Short stature was defined as a standing body height below the 5^th^ percentile using Center for Disease Control and Prevention’s (CDC) growth charts, corrected for age and gender. Obesity, overweight and underweight were defined as having a BMI greater than or equal to the 95^th^ percentile, greater than or equal to the 85^th^ percentile but less than the 95^th^ percentile, and less than 5^th^ percentile respectively using the CDC’s growth charts, corrected for age and gender.

Additionally, the sampling locations were studied from a socioeconomic point of view. The following indicators: education, income, healthcare and type of sewage system as an indicator of housing amenities, were assessed.

## Statistical analysis

For each subject, a Z-score was calculated for each anthropometric value, including the weight, height, and BMI. An SAS script was used to calculate the Z-scores using the ‘LMS’ method, where:

$$ Z=\frac{\left[{\left(\frac{Value}{M}\right)}^L\right]-1}{L\times S},\ L\ne 0 $$

In the above equation, ‘value’ corresponds to the subject’s BMI, weight, or height. The L, M, and S values correspond to parameters extracted from the 2000 CDC percentile data tables and can vary according to the child’s sex, age, or height. Percentiles were then calculated from the corresponding Z-scores. Using the same method, median Z-scores for each region, age and gender were calculated using the 2007 World Health Organization (WHO) data tables, and those values were compared with median Z-scores calculated using the CDC’s 2000 data tables.

Pearson's Chi-Squared testing was performed to assess for differences in prevalence of SS, obesity, overweight, and underweight between regions, school stratum and gender, with *p* <0.05 being used to define significance.

## Results

**Fig. 3:**
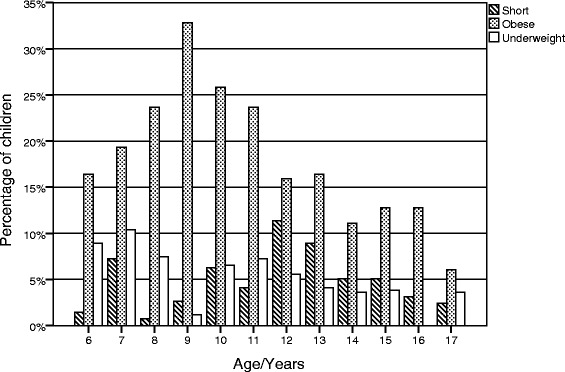
The prevalence of short stature, underweight, and obesity by age

A total of 2702 school students aged 6–17 years were enrolled, of which 1328 (49.2 %) were males and 1374 (50.8 %) were females. The median age was 11.2 ± 3.7 years. The demographic distribution of the sample is illustrated in Fig. 3. The overall prevalence of SS in Jordan was 4.9 % (*n* = 133), with a larger proportion being found in Southern regions compared to the Northern and Central regions (7.0 %, 5.3 % and 3.4 % respectively, *P*-value <0.001) (Table 2). On the other hand, there were no significant differences in the prevalence of SS among gender or school stratum.

**Table 2:** Prevalence of short stature, underweight, overweight, and obesity

	Short stature^a^		Underweight^b^		Overweight^c^		Obese^d^	
Characteristics	*n* (%)	*p*-value	*n* (%)	*p*-value	*n* (%)	*p*-value	*n* (%)	*p*-value
Region								
North	45 (5.3)	<0.001	55 (6.5)	0.04	149 (17.5)	0.21	121 (14.2)	0.021
Center	39 (3.4)		53 (4.6)		188 (16.3)		208 (18.1)	
South	49 (7.0)		46 (6.6)		126 (18.0)		76 (10.8)	
School stratum								
Public	70 (5.1)	0.71	80 (5.8)	0.69	215 (15.6)	0.047	129 (9.4)	<0.001
Private	63 (4.8)		74 (5.6)		248 (18.7)		276 (20.8)	
Gender								
Male	68 (5.1)	0.61	82 (6.2)	0.15	203 (15.3)	0.018	251 (18.9)	<0.001
Female	65 (4.7)		72 (5.2)		260 (18.9)		154 (11.2)	

^a^ Short stature defined as height-for-age <5^th^ percentile

^b^ Underweight defined as BMI-for-age <5^th^ percentile

^c^ Overweight defined as BMI-for-age ≥85^th^ percentile and < 95^th^ percentile

^d^ Obesity defined as BMI-for-age ≥95^th^ percentile

Abbreviations: *BMI* body mass index, *CDC* Centers for Disease Control and Prevention, *n* number

The overall prevalence of underweight subjects was 5.7 %. The rates were similar among both genders and both school strata. However, it was found that the Central region had a lower prevalence of 4.6 % compared to the Southern and Northern regions where the prevalence was 6.6 and 6.5 %, respectively (*P*-value = 0.04).

Obesity was recorded among 15.7 % of the total sample and was most prevalent in Central towns with 18.1 % of children crossing the cut-offs detailed in the table for BMI for age. A higher prevalence of obesity was also found in children enrolled in private schools with 20.8 % found to be obese compared to 9.4 % in public schools (*P*-value <0.001). Likewise, a higher prevalence of obesity was found among males, with 18.9 % being obese compared to 11.2 % of females (*P*-value <0.001). Furthermore, the Central region had a higher prevalence than either the Northern or the Southern region (18.1 %, 14.2 %, 10.8 %, respectively, *P*-value = 0.021).

In contrast to obesity, the overall prevalence of overweight was 17.3 % and it was not significantly associated with the region (*P*-value = 0.21), and females were more likely to be overweight compared to males (18.9 and 15.3 %, respectively, *P*-value = 0.018). Additionally, the prevalence of overweight children was higher in private schools as compared to public ones (18.7 %, 15.6 %, respectively, *P*-value = 0.047).

Overall, the median Z-scores for the whole sample were above reference values for height-, weight-, and BMI-for-age in both genders, according to the CDC and WHO references (Table 3). Male subjects had a higher than reference median Z-scores compared to their female counterparts in each of the three categories. Moreover, it was found that both the CDC and WHO references were in agreement to each other for median Z-scores for height-for-age in females with scores of 0.16 and 0.15 respectively. For males, however, the CDC and WHO references were 0.33 and 0.43, respectively. Median female Z-scores were also closest to mean reference values beyond the age of 10 and 12 years for height-for-age in WHO and CDC charts, respectively. However, no similar pattern was found among males. Regarding BMI-for-age, median Z-scores in both CDC and WHO references had a progressive increase from age 6–10 years in males, followed by a progressive decline from age 11–15. Females, however, had no consistent pattern across the array of ages.

**Table 3:** Comparison of median Z-scores with the 2000-CDC and the 2007-WHO growth charts

	Median Z-scores											
	Height for age				Weight for age				BMI for age			
	CDC		WHO		CDC		WHO		CDC		WHO	
Age	M	F	M	F	M	F	M	F	M	F	M	F
*All*	*0.33*	*0.16*	*0.43*	*0.15*	*0.73*	*0.42*	*0.83*	*0.71*	*0.64*	*0.39*	*0.84*	*0.53*
6	0.71	0.58	0.61	0.55	0.52	0.65	0.61	0.68	0.22	0.63	0.27	0.49
7	0.37	0.60	0.37	0.75	0.58	0.48	0.68	0.60	0.46	0.50	0.50	0.50
8	0.69	0.81	0.82	0.91	0.94	0.68	1.11	0.91	0.79	0.23	0.88	0.33
9	0.21	0.51	0.38	0.62	0.73	1.01	0.94	1.31	1.11	1.23	1.23	1.48
10	0.18	0.15	0.33	0.09	1.02	0.48	1.21	0.77	1.22	0.61	1.43	0.77
11	0.19	0.17	0.26	0.09	0.82	0.59	-	-	1.12	0.69	1.42	0.81
12	0.11	0.06	0.10	0.05	0.66	0.32	-	-	0.88	0.37	1.07	0.41
13	0.48	0.05	0.53	0.14	0.70	0.60	-	-	0.71	0.59	0.87	0.62
14	0.24	0.04	0.34	0.01	0.55	0.45	-	-	0.67	0.58	0.75	0.54
15	0.11	0.03	0.08	0.04	0.59	0.37	-	-	0.51	0.44	0.59	0.37
16	0.15	0.03	0.19	0.02	0.49	0.32	-	-	0.68	0.40	0.82	0.35
17	0.06	0.05	0.09	0.08	0.38	0.33	-	-	0.32	0.21	0.44	0.35

*Abbreviations: BMI* body mass index, *CDC* Centers for Disease Control and Prevention, *F* female, *M* male, *WHO* World Health Organization

As far as the socioeconomic status of the sampling locations, Table 4 provides basic summary statistics of the included cities and towns based on some socioeconomic indicators. Those include education, income, healthcare and type of sewage system as an indicator of housing amenities. The provided indicators showed that Southern regions; mainly rural, have lower socioeconomic status compared with Central and Northern regions, which are more urbanized. This is reflected by the obvious differences in the number of tertiary and secondary care hospitals, private universities, income and type of sewage system. (Table 4) [16].

**Table 4:** Some Socioeconomic Indicators of the Studied Regions^a^

Region	City/Town	Household member^b^ x1000	Public Schools	Private Schools	Public Univ.^c^	Private Univ.^c^	Tertiary Hosp.^d,e^	Secondary Hosp.^d,e^	% Households with annual income >6000 JDs^f^	Sewage System (Cesspool)
North	Irbid	1134	509	429	2	1	5	12	17.8	36
	Mafraq	300	481	43	1	0	0	4	4.4	^h^
	Jarash	190	154	42	0	1	0	2	3.2	^h^
	Ajlun	147	98	41	0	1	0	1	2.9	^h^
Center	Amman	2449	605	953	2	13	10	40	38.8	20
	Salt	426	181	135	1	0	0	2	5.7	43
	Zarqa	947	279	243	1	1	1	7	15.5	14
	Madaba	160	99	41	1	1	0	3	3.5	45
South	Karak^g^	248	197	52	1	0	0	0	2.8	92
	Tafilah^g^	89	89	16	1	0	0	0	1.4	66
	Ma’an^g^	121	141	22	1	0	0	0	1.7	50
	Aqaba^g^	138	70	31	1	0	0	0	2.7	14

^a^Jordanian Departement of Statistics, http://web.dos.gov.jo/sectors/economic, 2013

^b^Household members were approximated to the closest 1000

^c^Univ. = Universities

^d^Hosp. = Hospitals

^e^The number of primary care hospitals were not included in the table

^f^JDs = Jordanian Dinar

^g^The data of these towns include also their surroundings

^h^No available data for these towns

## Discussion

In this cross-sectional, nation-wide study, it was found that, among children and adolescents in Jordan, 4.9 % were of short stature, 5.7 % were underweight, 17.3 % were overweight, and 15.7 % were obese. Moreover, it was demonstrated that gender and the type of school attended were significantly associated with different prevalence rates of obesity and overweight. Additionally, rates of short stature, obesity, and underweight differed among the 3 different regions.

It was found that SS was most prevalent in rural provinces, with the Southern region having the highest prevalence, followed by the Northern region, and the Central region (7.0 %, 5.3 %, 3.4 %, respectively; *p* value <0.001). Gender, on the other hand, was not associated with the prevalence of SS; 5.1 % of the males had SS compared to 4.9 % of the females. Those gender-based prevalence rates lie within what has been reported in literature. The ‘Pro Child’ study, which spanned 9 European countries, found lower prevalence rates of short stature of 1.4 and 2.8 % in 11 year old males and females, respectively [17]. Another study from the West Bank, which neighbors Jordan, reported rates of 9.2 and 7.3 % among 13–15 year old males and females, respectively [18]. Underweight showed similar patterns of distribution to SS, with the Southern region again having the highest prevalence, followed by the Northern and Central regions (6.6 %, 6.5 %, 4.6 %, respectively, *P*-value = 0.04). Additionally, the prevalence was not associated with gender, with 6.5 % of males being underweight compared to 5.2 % of females (*P*-value = 0.15).

One possible explanation for the regional distribution patterns of short stature and underweight could lie in the effect socioeconomic status has on both of these conditions. It could be argued that the socioeconomic status of an individual has a major impact on the nutritional status and dietary habits of that individual, with suboptimal nutrition being more commonly expected among lower socioeconomic classes. Suboptimal nutrition is also expected to adversely affect health and growth. The effect of socioeconomic status can be further accentuated by the disparity of health and health care between areas of differing average income per household, as is the case between rural and urban areas, at least in Jordan. The Southern regions of Jordan are predominantly rural compared to the more affluent, urbanized Central region. Additionally, there was no significant difference in the prevalence rate of SS and underweight among the two genders. Similar findings regarding gender differences in SS were reported in studies conducted in neighboring countries. One study in Saudi Arabia reported no significant difference in the prevalence of SS between boys and girls (5–17 years of age) [19]. Another study in Ankara, Turkey investigated the prevalence of SS in 7–15 years school-aged children and also reported no gender differences regarding SS prevalence [20]. On the contrary, our findings are in contrast with two governorates from the West Bank; Ramallah and Hebron, in which prevalence of SS and underweight was higher among boys than girls [18]. Sharing a similar socioeconomic status as Jordan might be one reason why gender differences in regards to SS in Saudi Arabia and Turkey were consistent with our findings. However, socioeconomic status in the West Bank might differ when compared to Jordan’s due to a considerable geopolitical instability in that region. Furthermore, the data pointed to Jordan having a higher prevalence of SS than European countries [17], which enjoy a better standard of living, and lower than the West Bank [18], probably due to the reason mentioned above.

Previous studies conducted in China reported large discrepancies between rural and urban areas both in health conditions and in health care [21]. Despite improved children growth in all economic groups, a large growth disparity still exists between those Chinese rural and urban areas [22], and among the different economic subgroups within them [23, 24]. Additionally, several other reports shed some light on the contribution malnutrition has on the development of SS in the setting of socioeconomic difficulty. One study reported malnutrition as the third most common cause of SS [25], accounting for 9.8 % of the cases in Pakistan [25, 26]. In Brazil improving average socioeconomic status between 1975 and 2007 led to a reduction in the prevalence of SS by more than 80 % (from 37.1 to 7.1 %) [27]. Another study from Saudi Arabia found higher prevalence in the incidence of SS in the Southwestern region of Saudi Arabia compared to the rest of the country, citing the lower socioeconomic status of the area and its higher prevalence of malnutrition as possible causes [19]. A recent study conducted in England concluded that social inequalities reflected on the height of the children included in the study [28].

Another possible factor contributing to the patterns of SS and underweight distribution in our data could be the rate of consanguineous marriages in the Southern region, which is estimated to represent 35–55 % of all marriages in rural areas of Jordan [29, 30]. Consanguinity is associated with an increased probability of the propagation of recessive traits and diseases, some of which may adversely affect height and weight of children. For example, familial isolated growth hormone deficiency is one cause of SS with recessive inheritance patterns. A recent study which was conducted in Jordan among school children 6–16 years of age indicated that the prevalence of isolated growth hormone deficiency was significantly higher among short children of consanguineous marriage across all levels of consanguinity (86 %) than among the offspring of unrelated parents (50 %) [*P* = 0.001] [31]. Another study investigating the effect of inbreeding on the heights and weights of school children aged 5–15 years in Jammu (North India), reported that the mean difference in height, weight and BMI was −7.32 cm, −6.59 kg and −2.13 kg/m^2^ respectively for children of inbred families compared to those of non-inbred families [32].

A possibly interesting observation lies in that the prevalence of SS was highest among 12 year olds and lowest among 8 year olds. Compared to the Z-scores of the CDC and WHO growth charts, our sample’s height-for-age Z-scores for age 12 were significantly higher in males and females. However, for age 8, those values were close to the CDC’s and WHO’s values. One possible explanation for why the rates for SS were the highest at age 12 could be concerned with the national average age for onset of puberty. A previous unpublished study by A. Zayed pointed out that the average age of onset of puberty for the youth in Jordan was 14.2 years for males and 13.4 years for females. Compared to global averages, these figures are late. For example, delayed puberty in the US is defined as lack of signs of pubertal development by the age of 14 years for males and 13 years for females [33]. A delayed onset of puberty might also delay the pubertal growth spurt.

On the other end of the spectrum, it was found that 15.7 % of the studied subjects were obese, while 17.3 % were overweight. Analysis by gender showed that obesity was more prevalent among males, with 18.9 % being obese compared to 11.2 % of the females (*P*-value < 0.001). These figures and gender patterns appear to be similar to what was reported in literature. In one study performed in the United States, it was found that, in 2009–2010, 16.9 % of those aged 2–19 years were obese while 14.9 % were overweight. Further, it was noted that, in the same sample, the prevalence of obesity among males (18.6 %) was significantly higher than that among females (15.0 %) [34]. Figures in European countries, as reported by the ‘Pro Child’ study, appear to be lower than those reported in the United States, though the same pattern in gender difference persists; 5.6 % of the males included were obese while only 2.9 % of the females were so [17]. Another study conducted in Qatar that followed the International Obesity Taskforce’s (IOTF) guidelines found that 28.6 % of males aged 12–17 years were obese, while 7.6 % were overweight; compared to 18.6 and 4.7 %, respectively, of their female peers [35]. These differences in gender patterns could be explained through the effect gender identity has on body-image and how that is related to self esteem, with females being more likely to place a greater value on self body-image. One study found that adolescent females were more likely to associate body dissatisfaction with the concept of self-esteem [36]. Another study investigated the relationship between socioeconomic status, weight, age, gender, and body image in school children aged 6–19 years in New South Wales [37]. Their data indicated that the relationship between gender, body image and weight was significant in regards to overweight females being more likely than overweight males to report being ‘too fat’ (58.0 % vs. 36.5 %), while overweight males were more likely than their counterpart females to report their weight as ‘about right’ (59.0 % vs. 40.5 %) [37]. Moreover, the same study has also shown that overweight females were more likely to skip breakfast as well as be advised by others to lose weight (52.5 %) in comparison to overweight males (48.0 %) [37].

The discrepancy in health and health care between rural and urban areas seems to, again, influence the prevalence of obesity and overweight. In the data, this was reflected by a significantly higher prevalence of obesity in the urbanized Central region compared to the more rural Southern and Northern regions (18.1 %, 10.8 %, 14.2 %, respectively; *P*-value = 0.021). This trend has also been shown in a study conducted in China in 2010 in which the combined prevalence of overweight and obesity was found to be highest among urban boys (23.2 %), followed by rural boys (13.8 %), urban girls (12.7 %) and rural girls (8.6 %) [38]. It could be argued that despite the improvement in basic health infrastructure and health care access brought about by urbanization, the latter has also brought about changes in diet and lifestyle that might adversely affect health. These include the popularization of calorically dense food items and snacks and the tendency towards a sedentary lifestyle with the widespread use of automobiles, public transport services, and indoor entertainment media such as personal computers and televisions [39].

However, unlike the trends in SS and underweight prevalence, the data suggested an association between school strata and the rates of obesity and overweight. Among those attending private schools, 21.8 % were obese and 18.7 % were overweight, compared to 9.4 and 15.6 %, respectively, of their peers in public schools (*P*-value < 0.001 for the obese group comparison, and *p* < 0.047 for the overweight group comparison). This might point out to socioeconomic factors having an influence yet again; students attending private schools are more likely to come from more affluent backgrounds. Additionally, rural areas are more likely to have more public schools, where the tuition is offered for free, than private schools (Table 1). Of note, the tuition per one student in private schools ranges between 3000 and 20,000 JDs.

This study was the first, to the best of the authors’ knowledge, to attempt at discerning the prevalence of SS in Jordan. Additionally, it was the first to investigate the prevalence of obesity, overweight, and underweight on a national scale in Jordan. However, results were limited by the cross-sectional nature of the study, which precluded any effort to study the causality between variables.

## Conclusions

In conclusion, this study outlined the differences in weight and height trends between genders and between different regions in Jordan. Our data showed that there was an increased prevalence of SS and underweight in rural areas. This might be attributed to low socioeconomic status and high rate of consanguineous marriages in those regions. On the contrary, higher socioeconomic status in urbanized regions might contribute to higher rates of obesity and overweight there. Further research in this subject is needed, with an emphasis on pinpointing and stratifying the underlying reasons for pervasive weight and height patterns, especially in association with differences between regions. Plans to handle these health conditions will have to take these differences into consideration. In addition, improvements in socioeconomic status and counseling regarding consanguineous marriages might aid in reducing the prevalence of SS and underweight.

## Abbreviations

BMI: body mass index
CDC: Centers for Disease Control and Prevention
IOTF: International Obesity Taskforce
SAS: Statistical Analysis System
SS: short stature
WHO: World Health Organization
